# The Nup98 Homolog APIP12 Targeted by the Effector AvrPiz-t is Involved in Rice Basal Resistance Against *Magnaporthe oryzae*

**DOI:** 10.1186/s12284-017-0144-7

**Published:** 2017-02-15

**Authors:** Mingzhi Tang, Yuese Ning, Xiaoli Shu, Bo Dong, Hongyan Zhang, Dianxing Wu, Hua Wang, Guo-Liang Wang, Bo Zhou

**Affiliations:** 1 Institute of Nuclear-Agricultural Sciences, Zhejiang University, Hangzhou, China; 2 Institute of Virology and Biotechnology, Zhejiang Academy of Agricultural Sciences, Hangzhou, China; 3 Institute of Plant Protection, Chinese Academy of Agricultural Sciences, Beijing, China; 4 Department of Plant Pathology, the Ohio State University, Columbus, 43210 USA; 5 The Division of Genetics and Biotechnology, International Rice Research Institute, Los Baños, 4031 Philippines

**Keywords:** *APIP12*, *AvrPiz-t*, Protein-protein interaction, Blast resistance, Nup98

## Abstract

**Background:**

The effector *AvrPiz*-*t* of *Magnaporthe oryzae* has virulence function in rice. However, the mechanism underlying its virulence in host is not fully understood.

**Results:**

In this study, we analyzed the function of AvrPiz-t interacting protein 12 (APIP12) in rice immunity. APIP12 significantly bound to AvrPiz-t and APIP6 in its middle portion and N-terminus, respectively, in yeast two-hybrid assay. Glutathione S-transferase (GST) pull-down assay further verified the interactions of APIP12 with AvrPiz-t and APIP6. *APIP12* encodes a homologue of nucleoporin protein Nup98 without the conserved domain of Phe-Gly repeats and has no orthologue in other plants. Both knockout and knockdown of *APIP12* caused enhanced susceptibility of rice plants to virulent isolates of *M. oryzae*. The expression of some pathogenesis-related (*PR*) genes was reduced in both knockout and knockdown mutants, suggesting that *APIP12* is required for the accumulation of transcripts of *PR* genes upon the infection. It is worth noting that neither knockout/knockdown nor overexpression of *APIP12* attenuates *Piz-t* resistance.

**Conclusions:**

Taken together, our results demonstrate that *APIP12* is a virulence target of *AvrPiz-t* and is involved in the basal resistance against *M. oryzae* in rice.

**Electronic supplementary material:**

The online version of this article (doi:10.1186/s12284-017-0144-7) contains supplementary material, which is available to authorized users.

## Background

Plants possess a two-layer innate immune system to protect themselves from most microbial pathogens (Dodds and Rathjen 2010; Jones and Dangl 2006). The first layer of innate immunity is called PAMP-triggered immunity (PTI) that the recognition of pathogen-associated molecular patterns (PAMPs) is mounted by the pattern recognition receptors (PRRs) (Jones and Dangl 2006). PTI comprises the accumulation of reactive oxygen species (ROS) and the deposition of phenolic compounds (Jones and Dangl 2006). However, some pathogens are able to suppress the first layer of resistance by the secretion of effector proteins to target PRRs or key components in the PTI signaling pathway. Plants have evolved the second layer of the immune system called effector-triggered immunity (ETI), which is activated upon recognition of pathogen-secreted avirulence (Avr) effectors by cognate plant resistance (R) receptors (Bonardi and Dangl 2012). The resistance mediated by ETI is often associated with a hypersensitive response (HR) at the infection site to inhibit pathogen proliferation (Chisholm et al. 2006; Jones and Dangl 2006). Effectors are highly variable among different strains of a pathogen species. Thus, compared with PTI, ETI is more specific and assumed to be less durable.

The interaction between rice and *Magnaporthe oryzae*, the fungal pathogen causing the devastating rice blast disease, has been considered as one of the model phytopathosystems for understanding PTI and ETI in plant-fungal interactions (Dean et al. 2012; Liu et al. 2013). Over 25 rice *R* genes to blast have been molecularly characterized and most of them encode R proteins containing nucleotide binding site (NBS) and leucine-rich repeats (LRR) domains (Leung et al. 2015). In parallel with the advances in characterization of host *R* genes, over 10 *Avr* genes cognate to rice *R* genes have been characterized in *M. oryzae* (Wu et al. 2015; Zhang et al. 2015b). Two distinct models have been illustrated for the recognition of rice R proteins to their cognate *M. oryzae* Avr proteins. In the case of Pita/AvrPita, Pik/AvrPik, Pi-CO39/Avr1-CO39, and Pia/AvrPia, R proteins physically bind to their cognate Avr proteins (Cesari et al. 2013; Dangl and Jones 2001; Kanzaki et al. 2012). On the contrary, no direct interactions has been either identified or documented in other R/Avr pairs, e.g., no binding activity between Pi9 and AvrPi9 observed in a yeast two-hybrid (Y2H) assay (Wu et al. 2015). In the case of indirect interactions, recognition between R and Avr proteins is assumed to be activated by either so-called “guardees” or “decoys” (van der Hoorn and Kamoun 2008). In this regard, functional characterization of the host targets operated by *Avr* genes is vital for the elucidation of the recognition between *R* and *Avr* genes and the subsequent activation of resistance signaling.

We previously characterized the *AvrPiz-t* gene cognate to the rice blast *R* gene *Piz-t* using a map-based cloning strategy in *M. oryzae* (Li et al. 2009). Ectopic expression of *AvrPiz-t* in both tobacco and rice can suppress BAX-mediated cell death and PTI activated by both chitin and flg22, respectively, indicating that *AvrPiz-t* could primarily function as a virulence effector promoting pathogenicity without the *Piz-t* gene in the host (Li et al. 2009; Park et al. 2012). By Y2H screening, we identified 12 AvrPiz-t interacting proteins (APIPs) in rice. Functional characterization of APIP6 revealed that AvrPiz-t can suppress the E3 ligase activity of APIP6, which is required for the basal resistance to blast in rice (Park et al. 2012). In this study, we aim to characterize the function of APIP12 which encodes a protein homologous to nucleoporin 98 (Nup98). As essential components required for the assembly of nuclear pore complex (NPC), Nups play important roles in plant immunity. In Arabidopsis, both Nup160 and Nup96 were found to be involved in immunity-related mRNA export (Wiermer et al. 2012; Zhang and Li 2005). In this study, we will employ both Y2H and GST pull-down assays to validate and determine the regions required for direct binding between AvrPiz-t and APIP12. The interaction between APIP12 and other APIPs will also be tested to investigate whether different APIPs are functioning in a complex. The function of APIP12 in the basal and Piz-t-mediated resistance to rice blast will then be genetically characterized using knockout/knockdown mutants of APIP12.

## Results

### APIP12 encodes a unique protein sharing sequence and structure similarity to the Nup98 protein family

**Fig. 1:**
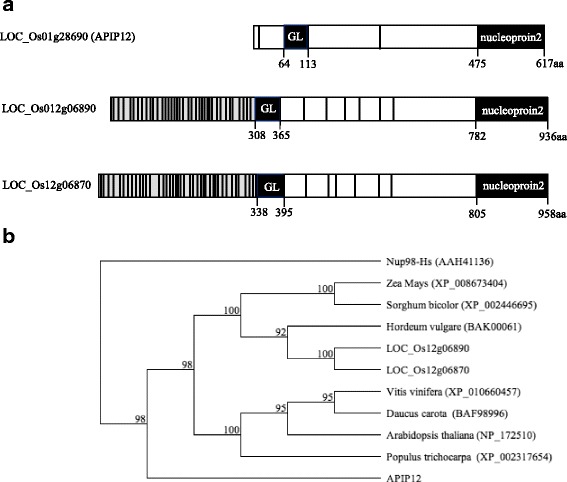
*APIP12* encodes a novel Nup98 homologue in rice. **a** The structure of three Nup98 homologues. The gray vertical bars represent FG repeats. The GLEBS and nucleoporin2 domains are indicated in filled rectangles. The “GL” refers to the GLEBS domain and the “aa” refers to the amino acid. The figure is drawn in scale. **b** The phylogenetic relationship of APIP12 and Nup98 orthologues in various plant species. The protein sequences are used for the multiple sequence alignment using Clustalw2 and the phylogenetic tree is viewed by Njplot (Tamura et al. 2007). The Nup98 in human (Nup98-Hs) is used as an outgroup

The cDNA clone of *APIP12* identified in a Y2H screen matched perfectly to the annotated rice gene model LOC_Os01g28690. The coding sequence (CDS) of *APIP12* was further cloned by RT-PCR from the seedlings of Nipponbare (NPB) and determined by sequencing. It is 1,854 bp in length and identical to the full-length cDNA clones (AK105445.1 and AK072730.1, http://cdna01.dna.affrc.go.jp/cgi-bin/sogo.cgi?pj=598&class=598&page=cDNA). The deduced protein product of *APIP12* contains two detectable domains, the N-terminal Gle2-binding sequence (GLEBS) and C-terminal nucleoporin2 domains (Fig. 1a). Two gene models, LOC_Os12g06870 and LOC_Os12g06890 arranged in a tandem array, were identified as the homologues of *APIP12* with significant sequence similarity in the rice genome. However, sequence similarity was found only in GLEBS and nucleoporin2 domains. For example, 54% (E-value: 5e^−14^) and 64% (E-value: 2e^−67^) identity in amino acid sequence in these two domains was identified between APIP12 and LOC_Os12g06890 (Fig. 1a). On the contrary, LOC_Os12g06890 or LOC_Os12g06890 share significant sequence similarity along the whole protein [79% identity in amino acid sequence (E-value: 0.0)]. Given the fact that both GLEBS and nucleoporin2 domains are conserved and featured in Nup98 family described in different organisms (Iwamoto et al. 2010), we therefore presume that *APIP12* encodes a homologous protein of Nup98. Intriguingly, APIP12 does not contain another conserved domain which is composed of repeats of two consecutive amino acid residues Phe-Gly (FG) in typical Nup98s (Fig. 1a) in human and other eukaryotes (Schmidt and Gorlich 2015). On the contrary, LOC_Os12g06870 and LOC_Os12g06890 contain FG domains surrounding the GLEBS domain (Fig. 1a). Phylogenetic analysis of homologues of Nup98 family in representative monocot and dicot plant species further revealed that all Nup98s including LOC_Os12g06870 and LOC_Os12g06890 are clustered together and distant from APIP12 (Fig. 1b). We thus speculate that LOC_Os12g06870 and LOC_Os12g06890 could represent the true orthologue of Nup98 in rice, which are likely derived from gene duplication from a common progenitor. Intriguingly, no APIP12 orthologues was identified in other plant species based on the extensive homolog search in the genomic sequence database at NCBI (http://www.ncbi.nlm.nih.gov, Fig. 1b). We thus postulate that APIP12 could represent a unique protein sharing sequence and structure similarity to Nup98 family. Nevertheless, it is elusive whether APIP12 functions in the NPC assembly as authentic Nup98s do in different organisms.

### APIP12 employs different regions to interact with AvrPiz-t and APIP6

**Fig. 2:**
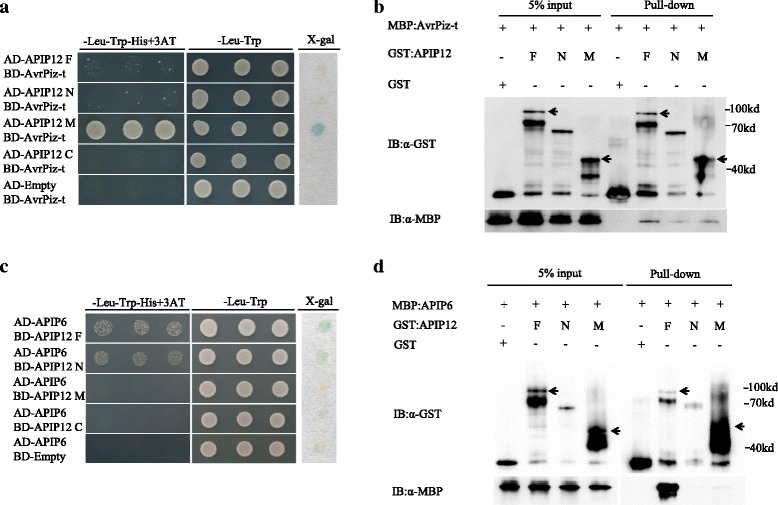
The physical binding of APIP12 with AvrPiz-t and APIP6. **a** The protein-protein interaction between AvrPiz-t and APIP12 in yeast two-hybrid assay. Four APIP12 fragments (F, N, M, C) were used for the test of the interaction with AvrPiz-t. The interactions between the tested proteins were assayed by monitoring yeast colonies growth on selective medium DOB-Leu-Trp-His to detect the activation of the His reporter gene. The LacZ reporter gene activity was detected for blue color of yeast colonies on a filter paper containing X-gal. **b** The protein-protein interaction between AvrPiz-t and APIP12 in GST pull-down assay. Different APIP12 fragments (F, N, M) each are fused with the GST tag and AvrPiz-t is fused with the MBP tag. The combination of GST- and MBP-fusion proteins are tested for the binding by immunoblotted using anti-GST (top panel) or anti-MBP (bottom panel) before (5% input) and after pull-down (Pulldown). GST was used as a negative control. The band corresponding to the expected size of the fusion protein is indicated in an arrow deduced based on the size of the protein ladder in the right. **c** The protein-protein interaction between APIP12 and APIP6 in yeast two-hybrid assay. **d** The protein-protein interaction between APIP12 and APIP6 in GST pull-down assay

To map the key regions of APIP12 required for its interaction with AvrPiz-t, we tested the binding activity using four different forms of APIP12 in a Y2H assay. These four APIP12 forms were full-length (APIP12F), N-terminus [APIP12N, position: 1–314 amino acid (aa)], middle portion (APIP12M, position: 315–475 aa), and C-terminus (APIP12C, position: 476–617 aa) which were created by referring to the region of nucleoporin2 domain (corresponding to 476–617 aa) and the sequence of the original *APIP12*’s cDNA clone identified in the Y2H screening for APIPs (corresponding to 313–617 aa). Positive signals, i.e., growth on selective medium and blue staining in X-gal assay, were only observed in the combination of APIP12M and AvrPiz-t but not in others (Fig. 2a). Therefore, we postulate that the middle portion of APIP12 is the major region binding to AvrPiz-t. Interestingly, this portion does not contain either GLEBS or nucleoporin2 domain. We also assume that N- and C-terminal portions could interfere with the binding between full-length APIP12 and AvrPiz-t in yeast without known reasons.

Next, we employed glutathione S-transferase (GST) pull-down assay to verify the interaction between APIP12 and AvrPiz-t in vitro. Immunoblotting analysis using the anti-GST and anti-MBP antibodies indicated a proper load of GST, GST-fused different portions of APIP12, and MBP-AvrPiz-t in the input (Fig. 2b). After pull-down procedure, the GST and 3 GST-fused APIP12s in the elution were able to be detected by the anti-GST antibody (Fig. 2b). Moreover, MBP-AvrPiz-t was detected in the elution from each of mixtures of GST-fused APIP12s and MBP-AvrPiz-t by anti-MBP antibody (Fig. 2b). On the contrary, no signal was detected in the one of GST incubates without APIP12 (Fig. 2b). These data clearly indicated that AvrPiz-t and APIP12 interacted to each other in GST pull-down assay. It is worth noting that the signal intensity of GST-APIP12s was much lower than the one of GST alone although equal amount of proteins were loaded in the input (Fig. 2b). Moreover, additional protein products with smaller sizes of GST-fused APIP12F and APIP12M displayed GST signal in both input and elution without a known reason (Fig. 2b).

To investigate whether APIP12 could form a complex with APIP6, we tested their interaction using both Y2H and GST pull-down assays. As Fig. 2c illustrated, positive signals were observed when either APIP12F or N was co-transformed with APIP6 (Fig. 2c). In GST pull-down assay, APIP6 was detected in the GST incubates of APIP12F (Fig. 2d). However, we could not detect the deposition of APIP6 in the GST incubates of APIP12N, displaying a distinct interaction manner from the one displayed in Y2H (Fig. 2c and d). In addition, we did not identify the interaction between APIP12M and APIP6 in either Y2H or pull-down assays, suggesting that APIP12M is not responsible for the binding of APIP12 to APIP6 (Fig. 2c and d). On the contrary, APIP12-M was found to be the major region binding to AvrPiz-t (Fig. 2a and b). These data prompted us to propose that APIP12 and APIP6 work as a protein complex involved in the basal resistance to rice blast.

The interaction between APIP12 and Piz-t was also investigated to check whether they can form complex by physical binding. Contrasting to the significant growth on the –Leu/-Trp medium, no growth was observed of the yeast cells co-expressed with different portions of Piz-t and APIP12 on the selective medium (Additional file 1: Figure S1). This data suggested that APIP12 might not physically bind to Piz-t.

### *APIP12* is involved in the basal resistance against rice blast

**Fig. 3:**
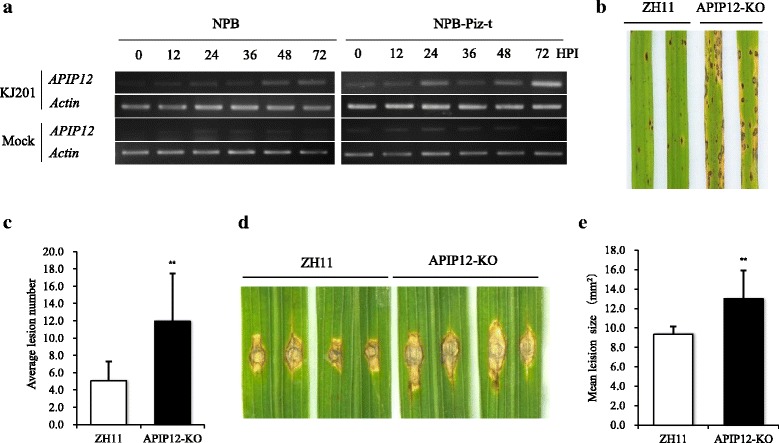
*APIP12* is required for the basal resistance to *M. oryzae* in rice. **a** Transcriptional pattern of *APIP12* in both compatible (NPB against KJ201) and incompatible (NPB-Piz-t against KJ201) interactions. The abundance of transcripts of *APIP12* is quantified at 0, 12, 24, 36, 48 and 72 h post inoculation (HPI) by semi-quantitative RT-PCR. Mock infection without pathogen is used as the control. *Actin* is used as an internal control gene. **b** Disease symptom of *APIP12* knockout mutant (APIP12-KO) and wild type (ZH11) after inoculation with *M. oryzae* isolate GUY11. Photograph of two representative leaves is taken 7 days after spray inoculation. **c** Quantification of lesion density in APIP12-KO and ZH11. The lesions at type 3 or above were counted in over 40 diseased leaves and the average lesion number was then calculated. Asterisks indicate statistically significant differences between APIP12-KO line and ZH11 (Student’s *t*-test, **P < 0.01). **d** Disease lesions in APIP12-KO and ZH11 in punch inoculation with *M. oryzae* isolate GUY11. Photograph of two representative leaves is taken 10 days after inoculation. **e** Quantitative analysis of disease lesions in APIP12-KO and ZH11 in punch inoculation. Error bars indicate standard deviation (SD) obtained from eight biological replicates (*n* = 8) and asterisks indicate statistically significant differences compared with ZH11 (Student’s *t*-test, ^**^ *P* < 0.01)

To elucidate the function of *APIP12* in disease resistance to rice blast, we firstly quantified the expression of *APIP12* in both compatible and incompatible interactions. As Fig. 3a illustrated, the transcripts of *APIP12* in both NPB (susceptible) and NPB-Piz-t (resistant) were accumulated gradually at different time points until 72 h after infection (HPI) of the strain KJ201 although the overall expression level of *APIP12* was relatively low. It is evident that the expression level of the control gene *actin* was indistinguishable (Fig. 3a). On the contrary, the expression of *APIP12* was not altered at different time points in the mock inoculation (Fig. 3a). These data indicate that *APIP12* is responsive to the rice blast infection in both incompatible and compatible interactions.

To functionally characterize *APIP12* in the basal resistance, a Tos17 insertion line, RMD_TosRS-04Z11BB04 (APIP12-KO) in rice variety ZH11 was identified and validated. The Tos17 is situated at 476-bp upstream from the start codon of *APIP12*, which was confirmed by the DNA analysis using specific primers (Additional file 2: Figure S2). RT-PCR analysis revealed that the expression of *APIP12* was undetectable in the insertion line. On the contrary, the wild type plant showed a weak but significant level of expression of *APIP12* (Additional file 2: Figure S2). We thus speculate that insertion of Tos17 successfully interrupts the gene expression of *APIP12*, providing an ideal genetic material for its functional characterization. Both ZH11 and APIP12-KO were inoculated with the virulent isolate GUY11. The mean lesion density in ZH11 was 5.07/3 cm^2^ whereas the one in APIP12-KO was 11.50/3 cm^2^ (Fig. 3b and c), indicating that disease symptom in the mutant plant is much severer than the one in wild type plant. In addition to the spray method, we also quantified the resistance of rice lines using the punch method. The mean lesion size in APIP12-KO was 12.98 mm^2^, which was significantly larger than the one of 9.37 mm^2^ in ZH11 (Fig. 3d and e). We further quantified disease resistance of *APIP12* knockdown (APIP12-KD) rice lines generated via RNAi. Compared to the one in the wild type plant, the expression of *APIP12* is significantly reduced in both APIP12-KD lines (46-2 and 49-2, Additional file 3: Figure S3a). The lesion size observed in these two KD lines was much larger than the one in the wild type plant, indicating that they are more susceptible to the virulent isolate (Additional file 3: Figure S3b and c). Taken together, we conclude that either knockout or knockdown of *APIP12* resulted in more susceptibility of rice plants to rice blast.

In addition to the knockout/knockdown mutants, 3 independent lines of *APIP12* overexpression (APIP12-OX-69, −70, and −73) validated by gene expression (Additional file 4: Figure S4a) were challenged with the virulent isolate KJ201 by both spray and punch inoculations. No significant difference in disease resistance was observed in APIP12-OX lines compared to the non-transgenic NPB plant (Additional file 4: Figure S4b), suggesting that overexpression of *APIP12* did not alter the resistance to rice blast.

### *APIP12* is not required for the *Piz-t*-mediate resistance to rice blast

**Fig. 4:**
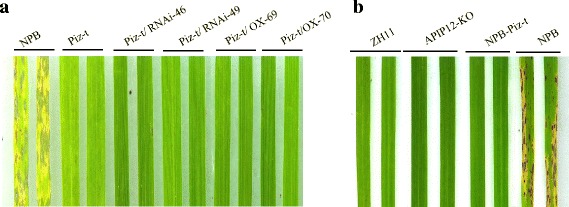
Ectopic expression of *APIP12* does not interfere with *Piz-t* mediated resistance to rice blast. **a** Reactions of Piz-t/APIP12-RNAi and Piz-t/APIP12-OX hybrid lines against the *Piz-t* avirulent isolate KJ201. **b** Reactions of different rice lines against the transgenic isolate GUY11-AvrPiz-t. Two representative leaves of each rice line were selected for taking the photograph 7 days after inoculation

**Fig. 5:**
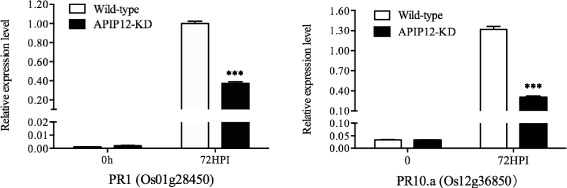
Expression levels of *PR1* (*Os01g28450*) and *PR10.a* (*Os12g36850*) in the APIP12-KD mutant and wild type plants after *M. oryzae* Inoculation. *Error bars* indicate the SD from three biological replicates (*n* = 3), and *asterisks* indicate statistically significant differences of APIP12-KD mutant compared with wild type plants (Student’s *t*-test, ***P < 0.001)

To further investigate whether *APIP12* is required for the *Piz-t*-mediated resistance, we evaluated the resistance of *Piz-t* transgenic plants with ectopic expression of *APIP12* against *Piz-t* avirulent isolates. Given the availability of different transgenic lines in the same NPB background, we generated fixed lines of Piz-t/APIP12-KD-46-2, Piz-t/APIP12-KD-49-2, Piz-t/APIP12-OX-69, Piz-t/APIP12-OX-70 by crossing between *Piz-t* transgenic lines and APIP12-KD and -OX lines. Disease assessment of these hybrid lines against the Piz-t avirulent isolate KJ201 revealed that they showed strong resistance as did NPB-Piz-t (Fig. 4a). On the contrary, NPB is susceptible to KJ201 (Fig. 4a). These data clearly indicated that either knockdown or overexpression of *APIP12* did not attenuate *Piz-t* mediated resistance to the avirulent strain. Coincidently, ZH11 is resistant to the strain GUY11-AvrPiz-t but susceptible to GUY11 (Figs. 3b and 4b), suggesting that it could contain *Piz-t*. Determination of the genomic sequence of the *Piz-t* homolog in ZH11 further confirmed the existence of *Piz-t* in ZH11 (data not shown). Thus, the APIP12-KO line provides another ideal rice line in a different genetic background to validate the function of *APIP12* in *Piz-t* triggered resistance. As illustrated in Fig. 5b, no disease lesion was observed in APIP12-KO to GUY11-AvrPiz-t. Furthermore, the resistant and susceptible reactions of NPB-Piz-t and NPB against GUY11-AvrPiz-t reiterated the specific recognition between *Piz-t* and *AvrPiz-t* (Fig. 4b). We thus postulate that the disruption of *APIP12* didn’t interfere with the *Piz-t/AvrPiz-t* mediated immunity in rice.

### The expression of *PR* genes is subdued in APIP12-knockout/knockdown mutants upon the infection of rice blast

As one of hallmarks of plant response to pathogen infection, the transcription of many pathogenesis-related (*PR*) genes is up-regulated. We thus compared the transcription of different *PR* genes between the APIP12-KO mutant and the wild type plant at 72 HPI. Fourteen out of 23 *PR* genes selected from 7 families displayed significantly lower levels of transcription in APIP12-KO than in ZH11 (Table 1). For example, the *PR10* gene family member, LOC_Os12g36850 in APIP12-KO had only 0.17 relative expression level of the one in ZH11. We selected several *PR* genes for the comparison of their expression level in both APIP12-KO and -KD mutants. As Additional file 5: Table S1 indicated, these *PR* genes displayed a reduced level of expression at 72 HPI in APIP12-KD mutant. However, no significant difference with respect to the expression reduction was observed between knockout and knockdown mutants (Additional file 5: Table S1). To further investigate the expression pattern of *PR* genes in both APIP12-KD mutant and wild type plant during the rice blast infection, a time-course analysis of gene expression of LOC_Os01g28450 (*PR1* family) and LOC_Os12g36850 (*PR10* family) was conducted. Each of them displayed a similar pattern of gene expression, which was up-regulated at 72 HPI in both mutant and wild type plant (Fig. 5). The expression level of both *PR* genes was significantly lower in APIP12-KO than the one in wild type plant at 72 HPI (Fig. 5). These results indicated that the interference with *APIP12* resulted in the subdued expression of some *PR* genes upon the challenge of rice blast.

**Table 1:** Relatively expression level of *PR* genes in the APIP12-knockout mutant (M) compared to the one in the wild type plant (W) at 72 h after infection (HPI) with the virulent isolate GUY11

*PR* gene family	Gene models	Relative expression level (M/W)
*PR1*	*Os01g28450*	0.45 ± 0.090***
	*Os01g28500*	0.53 ± 0.177**
	*Os05g51660*	0.93 ± 0.261
	*Os07g03279*	1.37 ± 0.440
	*Os07g03710*	0.60 ± 0.089**
*PR2*	*Os01g71340*	0.68 ± 0.018***
	*Os01g51570*	0.97 ± 0.105
*PR3*	*Os06g51060*	0.57 ± 0.055***
	*Os06g51050*	0.71 ± 0.089
	*Os10g39680*	0.63 ± 0.132**
*PR4*	*Os11g37950*	0.22 ± 0.116***
	*Os11g37960*	1.39 ± 0.218
	*Os11g37970*	0.51 ± 0.060***
*PR5*	*Os03g46070*	0.33 ± 0.043***
	*Os08g43510*	0.71 ± 0.324
	*Os12g43380*	0.65 ± 0.150
	*Os12g43440*	0.59 ± 0.189
*PR6*	*Os01g03360*	0.47 ± 0.102***
	*Os01g03340*	1.03 ± 0.604
*PR10*	*Os12g36830*	0.34 ± 0.080***
	*Os12g36850*	0.17 ± 0.033***
	*Os12g36860*	0.24 ± 0.038***
	*Os12g36880*	0.37 ± 0.051***

The data was normalized using Ubiquitin gene (*OsUG*) as an endogenous control and analyzed to calculate relative expression values using 2^−ΔΔCt^ method. Asterisks indicate significant differences between APIP12-knockout mutant and the wild type plant (Student’s *T*-Test, **: *P* < 0.01, ***: *P* < 0.001)

## Discussion

Multiple lines of evidence have demonstrated that phytopathogens secrete and deliver diverse effectors into plant cells to interfere with individual defense responses (Gohre and Robatzek 2008). It has been also clearly illustrated that in the absence of its cognate resistance protein, the so-called avirulence effector fulfills the virulence function to promote the pathogenicity of the pathogen (Dou and Zhou 2012). As described previously, *AvrPiz-t* can suppress the BAX-mediated cell death in tobacco leaves and the generation of reactive oxygen species (ROS) induced by PAMP elicitors in rice (Li et al. 2009; Park et al. 2012), demonstrating the virulence activity of *AvrPiz-t* in the absence of its cognate resistance gene *Piz-t*. Likewise, the RXLR effector AVR1 of *Phytophthora infestants* functions as a virulence factor that promotes colonization and suppresses callose deposition, a hallmark of basal defense in tobacco (Du et al. 2015). It has been extensively illustrated that many phytopathogen effector proteins execute the virulence activity through the manipulation of their host targets. For example, *Pseudomonas syringae* type III effector HopF2Pto targets *Arabidopsis* RIN4 protein to promote the growth of bacterium in plant (Wilton et al. 2010). The effector Avr3a essential for the virulence of *P. infestants* targets and stabilizes the plant E3 ligase CMPG1 for potentially preventing host cell death during the biotrophic phase of infection (Bos et al. 2010). The identification of 12 APIPs targeted by AvrPiz-t allows the dissection of how AvrPiz-t contributes its virulence functionality to rice blast pathogen. Park et al. (2012) demonstrated that AvrPiz-t could target APIP6 and suppress its E3 ligase activity during the infection process. Moreover, *APIP6* is required for PTI and its silencing leads to the compromised resistance to rice blast. These data provided the evidence that *AvrPiz-t* conveys its virulence advantage by manipulating *APIP6* that is involved in the basal resistance against rice blast. Most recently, the study on *APIP10*, which encodes another E3 ligase, elucidated a similar mechanism on how AvrPiz-t interacts with host target and promotes its virulence to rice blast (Park et al. 2016). Most recently, AvrPiz-t was demonstrated to suppress the transcriptional activity and protein accumulation of APIP5 for promoting the effector-triggered necrosis in rice (Wang et al., 2016).

In this study, we introduce APIP12, the 4^th^ host protein targeted by AvrPiz-t and attempt to illustrate its role in rice immunity against rice blast. *APIP12* displays an induced expression pattern in both compatible and incompatible reactions, suggesting that it responds positively to rice blast infection. Either knockout or knockdown of *APIP12* in rice led to the enhanced susceptibility to rice blast. On the contrary, the mutation of *APIP12* did not compromise the immunity to rice blast mediated by the pair of *Piz-t* and *AvrPiz-t*. These data indicated that *APIP12* is most likely not required for the *Piz-t*-mediated resistance. Nevertheless, it functions as a positive regulator in the basal resistance against rice blast. The attenuated induced expression of *PR* genes in the compatible interaction to rice blast in *APIP12* mutants further supported this hypothesis. It is thus reasonable to speculate that APIP12 is one of the virulence targets, like APIP6 and APIP10, and manipulated by AvrPiz-t for executing its virulence function in rice (Park et al. 2012; Park et al. 2016). Moreover, the interaction assay in yeast clearly indicated that APIP12 employed different domains to interact with AvrPiz-t and APIP6, providing a clue of the connection among these 3 proteins. However, it is still elusive how AvrPiz-t contributes its virulence to rice blast in rice through the modification of APIP12.

Increasing evidence points the notion that some components of NPC are involved in the regulation of immunity to diverse pathogens by selectively regulating the exchange of proteins and RNAs of components in plant defense signaling. For example, in the largest subunit of the NPC, 3 out of 9 putative complex members of the Nup107-160 sub-complex, i.e.*,* MOS3/Nup96, Nup160, and Seh1, were found to be essential for the basal resistance and snc1 activated autoimmunity in *Arabidopsis* (Du et al. 2009; Ratner et al. 2007). Given the importance of NUPs in disease resistance, we assume that they could also be targeted by pathogen effectors like those components in defense signaling from an evolutionary point of view (Gohre and Robatzek 2008). The existence of two Nup98 featured domains (GLEBS and nucleoporin2) in APIP12 promoted us to speculate that it is a component of NPC in rice. The interaction between APIP12 with OsNup96 (LOC_03g07580) was identified by Y2H assay (Additional file 6: Figure S5), providing additional evidence to the association between APIP12 with other components in NPC as characterized previously (Ratner et al. 2007). However, we found that APIP12 is present in the cytoplasmic foci rather than in the nuclei or the nuclear envelope to which typical NUPs are localized (Boeglin et al. 2016; Zhang and Li 2005). Moreover, distinct from other versions of Nup98s identified in all branches of eukaryotic life including rice containing the FG domain (Schmidt and Gorlich 2015), APIP12 does not contain this conserved domain. The FG domain was documented to be important for the binding to the constituents of the NPC scaffold. For example, the binding of the FG domain of Nup98 with Nup93 is important for the assembly of FG domain in the center of the NPC (Xu and Powers 2013). More importantly, the FG domain could mainly function in the formation of the selective permeability barrier of NPC by its spontaneous phase-separation from aqueous solution into FG particles (Robles et al. 2012; Xu and Powers 2013). Intriguingly, APIP12 is present only in rice but not in other monocot and dicot plants. Taken together, we postulate that APIP12 could represent a unique Nup98 homologue involved in the basal resistance to rice blast.

## Methods

### Plant materials and growth conditions

Rice (*Oryza sativa* L.) lines, including Nipponbare (NPB), Zhonghua11 (ZH11), the *Piz-t* transgenic line in NPB (NPB-Piz-t) as described previously (Li et al. 2009), are used in this study. A Tos17 insertion line of the *APIP12* gene in ZH11, TosRs_04Z11BB04, is obtained from the Rice Mutant Database (http://rmd.ncpgr.cn/). Rice plants are grown in pots and kept in a growth chamber ranging from 22 to 26 °C with the cycle of 14 h light/10 h dark.

### Plasmid construction and rice transformation

To generate the RNAi vector of *APIP12*, a 339-bp fragment of at the C-terminus (1,261 and 1,600 bp downstream of the start codon) was amplified and cloned into the pANDA vector (Miki and Shimamoto 2004) by LR gateway reactions. The *APIP12* overexpression vector was constructed in pCambia1301 in which the full-length coding sequence (CDS) of *APIP12* was inserted downstream of the maize (*Zea mays*) ubiquitin promoter. All the primers are given in Additional file 5: Table S1. The vectors were transformed into NPB to generate transgenic lines via the *Agrobacterium*-mediated rice transformation method described previously (Qu et al. 2006). Over 20 independent transgenic lines were generated and used for functional characterization.

### Rice blast inoculation and disease resistance evaluation

The *M. oryzae* isolates GUY11, KJ201, and GUY11-AvrPiz-t transformed strain (Li et al. 2009) were used for the rice blast inoculation. The fungus was cultured on complete medium (CM) for 2 weeks at 28 °C. Three-week old rice seedlings were inoculated with the conidial suspensions at concentration of 10-15×10^4^ spores ml^−1^ with a sprayer (Qu et al. 2006). Disease assessment was conducted 7 days after inoculation. The experiments were repeated for two times. In addition to the spray method, the punch method was employed to quantify the blast resistance of 6-week old plants as described previously (Ono et al. 2001). Mean size of lesions was measured using Image J software (https://imagej.nih.gov/ij/). The Student’s *T*-Test was used to determine the significance of differences between mutant and wild type.

### Phylogenetic analysis

To identify Nup98 homologues in different plant species, homolog search against the NCBI non-redundant (NR) protein database (http://blast.ncbi.nlm.nih.gov/) was performed using *APIP12* as a query. Accession numbers of the homologues are as follows: LOC_Os12g06870 and LOC_ Os12g06890 (*Oryza sativa*, identified from rice genome database: http://rice.plantbiology.msu.edu/), NP_172510 (*Arabidopsis thaliana*), BAF98996 (*Daucus carota*), XP_010660457 (*Vitis vinifera*), XP_002317654 (*Populus trichocarpa*), XP_008673404 (*Zea mays*), BAK00061 (*Hordeum vulgare*), and XP_002446695 (*Sorghum bicolor*). Protein sequences were aligned using the Clustalw2 program (http://www.ebi.ac.uk/Tools/msa/clustalw2/) with the default parameters. The phylogenetic tree was viewed by using the neighbor-joining method of the MEGA 4.1 software (Tamura et al. 2007). A bootstrap analysis with 1000 replicates was performed to test the confidence of topology. We included Nup98 in human as an outgroup (Accession No. : AAH41136) in the phylogenetic analysis.

### Yeast two-hybrid analysis

The Y2H assay was performed by following the procedure as described previously (Park et al. 2012) using the ProQuest Two-Hybrid system (Invitrogen, USA). The mature form of AvrPiz-t (residues 19–108) was cloned into pDBLeu-BD to generate the BD:Ns-AvrPiz-t bait construct. The full length, N-terminus, middle portion (M) and C-terminus of APIP12 were cloned into pPC86-AD to generate the AD:APIP12F, AD:APIP12N, AD:APIP12M and AD:APIP12C prey constructs, respectively. All the primers are given in Additional file 5: Table S1. Yeast cells with co-transformed pDBLeu- and pPC86-derived vectors were plated and incubated on synthetic medium lacking leucine, tryptophan (DOB-Leu-Trp) and the selective medium DOB-Leu-Trp-His (supplemented with 50 mM of 3-amino-1,2,4-triazole) at 30 °C for 3 days for the observation of cell growth to detect the His reporter gene activity. The yeast colonies gowning on DOB-Leu-Trp medium were blotted onto filter papers. The filter papers were frozen in liquid nitrogen for 1 min, and then were placed on top of a pre-soaked filter paper in a 100 mm sterile plate with Z buffer/X-gal solution [2 ml of Z buffer, 6 μl ofβ-mercaptoethanol, 20 μl of X-gal stock solution (40 mg/ml)]. The plate is placed in the incubator at 30 °C for 8 h to detect the LacZ reporter gene activity.

### GST pull-down assay

APIP12F, APIP12N and APIP12M fragments were cloned into pGex-6p-1 in frame with GST tag to generate GST-APIP12F, GST-APIP12N and GST-APIP12M constructs. The mature form of AvrPiz-t (Avrpiz-t^19-108^) and APIP6 were cloned into pMAL-c2 vector in frame with MBP to generate MBP-AvrPiz-t and MBP-APIP6 constructs. Details of primers are given in Additional file 5: Table S1. Both GST-tagged and MBP-tagged vectors were transformed and expressed in *E. coli* strain Rosetta2 (DE3). The GST pull-down assay was conducted by following the procedure described previously (Park et al. 2016). About 2 μg of MBP- and GST-fused proteins were incubated at 25 °C for 2 h. The protein mixture was then incubated with 50 μg of glutathione-agarose beads for 2 h. Then the mixture was washed six times each with 1 ml of ice-cold PBS buffer. The eluted proteins after pull-down procedure were resolved in 10% SDS-PAGE gel and proteins were then transferred to Immobilon-PSQ PVDF membrane (Millipore). Diluted anti-GST antibody (Roche, USA) and anti-MBP antibody (Biomol, USA) were used for immunoblotting analysis. Chemiluminescene was detected using Pierce ECL substrate (Promega, USA) and the ChemiDoc XRS system (Bio-Rad).

### Gene transcription analysis

Total RNAs were extracted from rice tissues using Trizol reagent by following the manufacturer’s instruction (Invitrogen, USA) and were then treated with DNase RQ1 (Promega, USA) to eliminate the contamination of genomic DNAs. Approximately 2 ug of total RNAs were used for reverse transcription using the Reverse Transcription System (Promega, USA). Semi-quantitative PCR was conducted with the procedure as described previously (Zhang et al. 2015a) to quantify the gene expression of *APIP12* at different time points after rice blast and mock infection. The rice *actin* gene was used as a control in semi-quantitative PCR. Quantitative PCR was carried out using SYBR Green Supermix (TaKaRa, Japan) on a CFX96 96-well real-time PCR unit (Bio-Rad). The CFX96 software was used to calculate threshold cycle values. The data was normalized using the *ubiquitin* gene (*OsUG*) as an endogenous control and analyzed to calculate relative expression values using 2^−ΔΔCt^ method (Livak and Schmittgen 2001). The two-tailed Student’s *T*-Test was used to determine the statistical significance. All the primers are listed in Additional file 7: Table S2. The expression analyses were repeated three times.

## Conclusions

We described that the rice APIP12 protein was one of the host targets of the Magnaporthe oryzae avirulence effector AvrPiz-t and showed significant sequence similarity to Nup98 likely involved in the assembly of NPC. Functional characterization of *APIP12* revealed that it was required for the basal resistance against rice blast. However, it was likely dispensable for the *Piz-t* mediated resistance.

## Additional files

Additional file 1: Figure S1. Interaction between APIP12 and Piz-t. (PPTX 380 kb)
Additional file 2: Figure S2. Molecular validation of *APIP12* knockout mutant. (PPTX 34 kb)
Additional file 3: Figure S3. Knockdown of *APIP12* by RNAi results in compromised resistance to rice blast. (PPTX 501 kb)
Additional file 4: Figure S4. Overexpression of *APIP12* does not alter the disease resistance to rice blast. (PPTX 117 kb)
Additional file 5: Table S1. Relatively expression level of *PR* genes in both APIP12-KD (RNAi) and APIP12-KO mutants compared to respective wild type plants at 72 h after infection with the virulent isolate GUY11. (DOC 30 kb)
Additional file 6: Figure S5. APIP12 interacts with Nup96 (LOC_Os03g07580) in Y2H assays. (PPTX 69 kb)
Additional file 7: Table S2. Primers used in this study. (DOC 69 kb)

## Abbreviations

APIP: AvrPiz-t interacting proteins
Avr: Avirulence effectors
ETI: Effector-triggered immunity
GFP: Green fluorescent proteins
GLEBS: Gle2-binding sequence
GST: Glutathione S-transferase
HPI: Hours after infection
HR: Hypersensitive response
LRR: Leucine-rich repeats
NBS: Nucleotide binding site
PAMPs: Pathogen-associated molecular patterns
PR: Pathogenesis-related
PRRs: Pattern recognition receptors
PTI: PAMP-triggered immunity
Y2H: Yeast two-hybrid
