# Supplementary material for: The Nup98 Homolog APIP12 Targeted by the Effector AvrPiz-t is Involved in Rice Basal Resistance Against Magnaporthe oryzae
Source: Rice (N Y). 2017 Feb 15;10:5. doi: 10.1186/s12284-017-0144-7 (PMC5311014; doi:10.1186/s12284-017-0144-7)
Supplement: Additional file 1: Figure S1. — Interaction between APIP12 and Piz-t. (PPTX 380 kb) [file 12284_2017_144_MOESM1_ESM.pptx]

## Slide 1
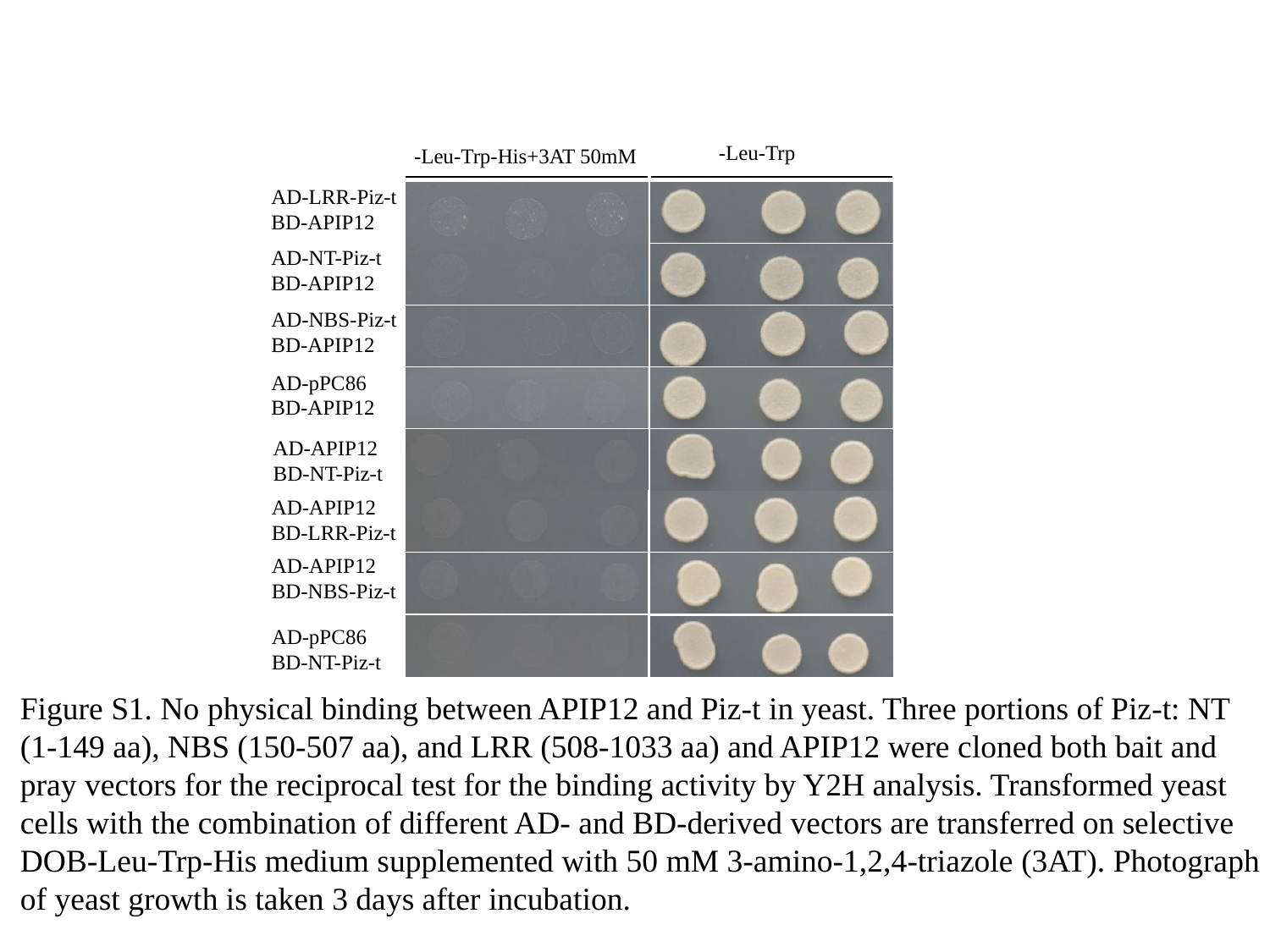

-Leu-Trp
-Leu-Trp-His+3AT 50mM
AD-LRR-Piz-t
BD-APIP12
AD-NT-Piz-t
BD-APIP12
AD-NBS-Piz-t
BD-APIP12
AD-pPC86
BD-APIP12
AD-APIP12
BD-NT-Piz-t
AD-APIP12
BD-LRR-Piz-t
AD-APIP12
BD-NBS-Piz-t
AD-pPC86
BD-NT-Piz-t
Figure S1. No physical binding between APIP12 and Piz-t in yeast. Three portions of Piz-t: NT (1-149 aa), NBS (150-507 aa), and LRR (508-1033 aa) and APIP12 were cloned both bait and pray vectors for the reciprocal test for the binding activity by Y2H analysis. Transformed yeast cells with the combination of different AD- and BD-derived vectors are transferred on selective DOB-Leu-Trp-His medium supplemented with 50 mM 3-amino-1,2,4-triazole (3AT). Photograph of yeast growth is taken 3 days after incubation.
