# Supplementary material for: The Nup98 Homolog APIP12 Targeted by the Effector AvrPiz-t is Involved in Rice Basal Resistance Against Magnaporthe oryzae
Source: Rice (N Y). 2017 Feb 15;10:5. doi: 10.1186/s12284-017-0144-7 (PMC5311014; doi:10.1186/s12284-017-0144-7)
Supplement: Additional file 2: Figure S2. — Molecular validation of APIP12 knockout mutant. (PPTX 34 kb) [file 12284_2017_144_MOESM2_ESM.pptx]

## Slide 1
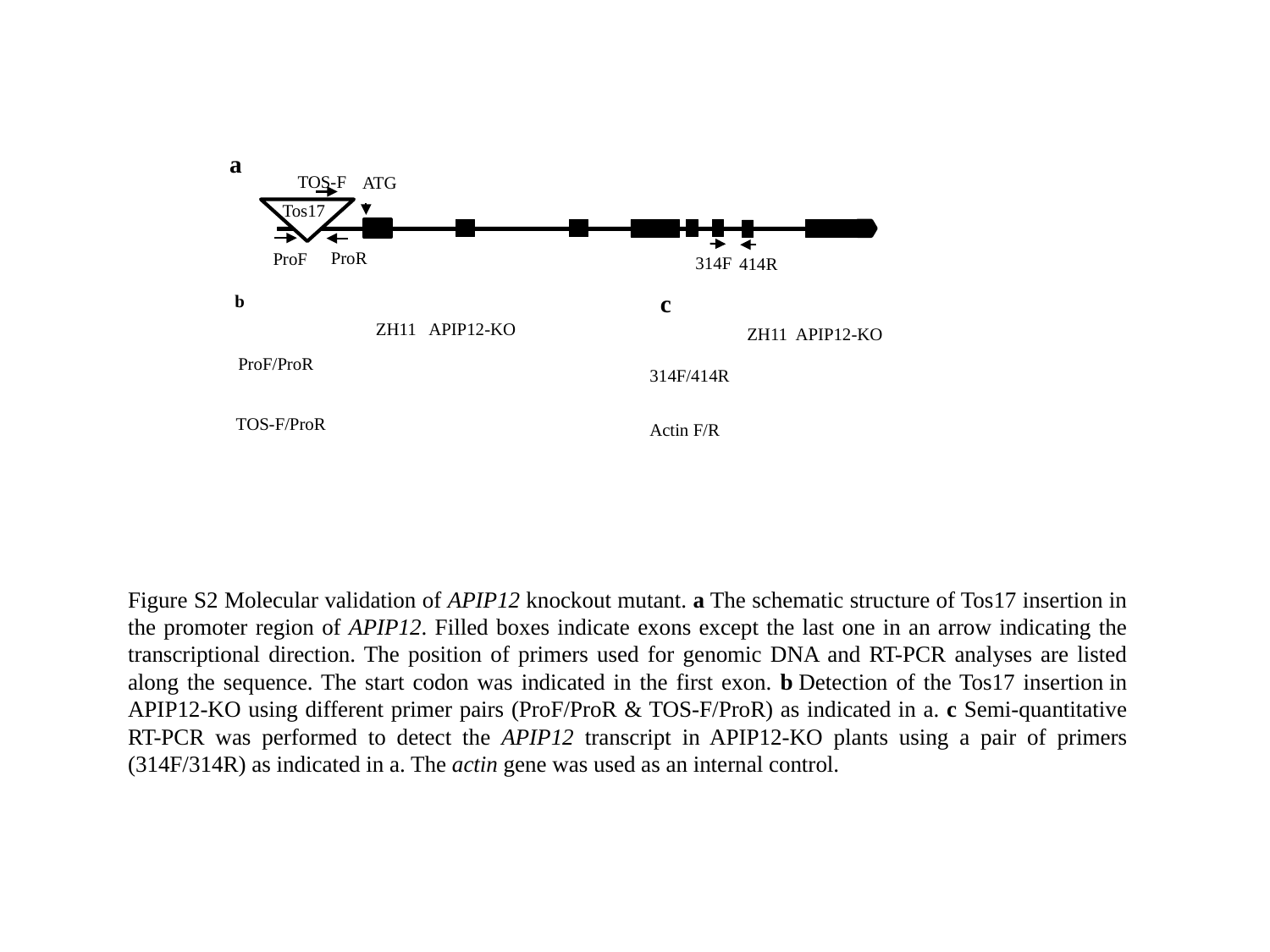

a
TOS-F
ATG
Tos17
ProR
ProF
314F
414R
c
ZH11 APIP12-KO
314F/414R
Actin F/R
b
ProF/ProR
TOS-F/ProR
 ZH11 APIP12-KO
Figure S2 Molecular validation of APIP12 knockout mutant. a The schematic structure of Tos17 insertion in the promoter region of APIP12. Filled boxes indicate exons except the last one in an arrow indicating the transcriptional direction. The position of primers used for genomic DNA and RT-PCR analyses are listed along the sequence. The start codon was indicated in the first exon. b Detection of the Tos17 insertion in APIP12-KO using different primer pairs (ProF/ProR & TOS-F/ProR) as indicated in a. c Semi-quantitative RT-PCR was performed to detect the APIP12 transcript in APIP12-KO plants using a pair of primers (314F/314R) as indicated in a. The actin gene was used as an internal control.
