# Supplementary material for: The Nup98 Homolog APIP12 Targeted by the Effector AvrPiz-t is Involved in Rice Basal Resistance Against Magnaporthe oryzae
Source: Rice (N Y). 2017 Feb 15;10:5. doi: 10.1186/s12284-017-0144-7 (PMC5311014; doi:10.1186/s12284-017-0144-7)
Supplement: Additional file 3: Figure S3. — Knockdown of APIP12 by RNAi results in compromised resistance to rice blast. (PPTX 501 kb) [file 12284_2017_144_MOESM3_ESM.pptx]

## Slide 1
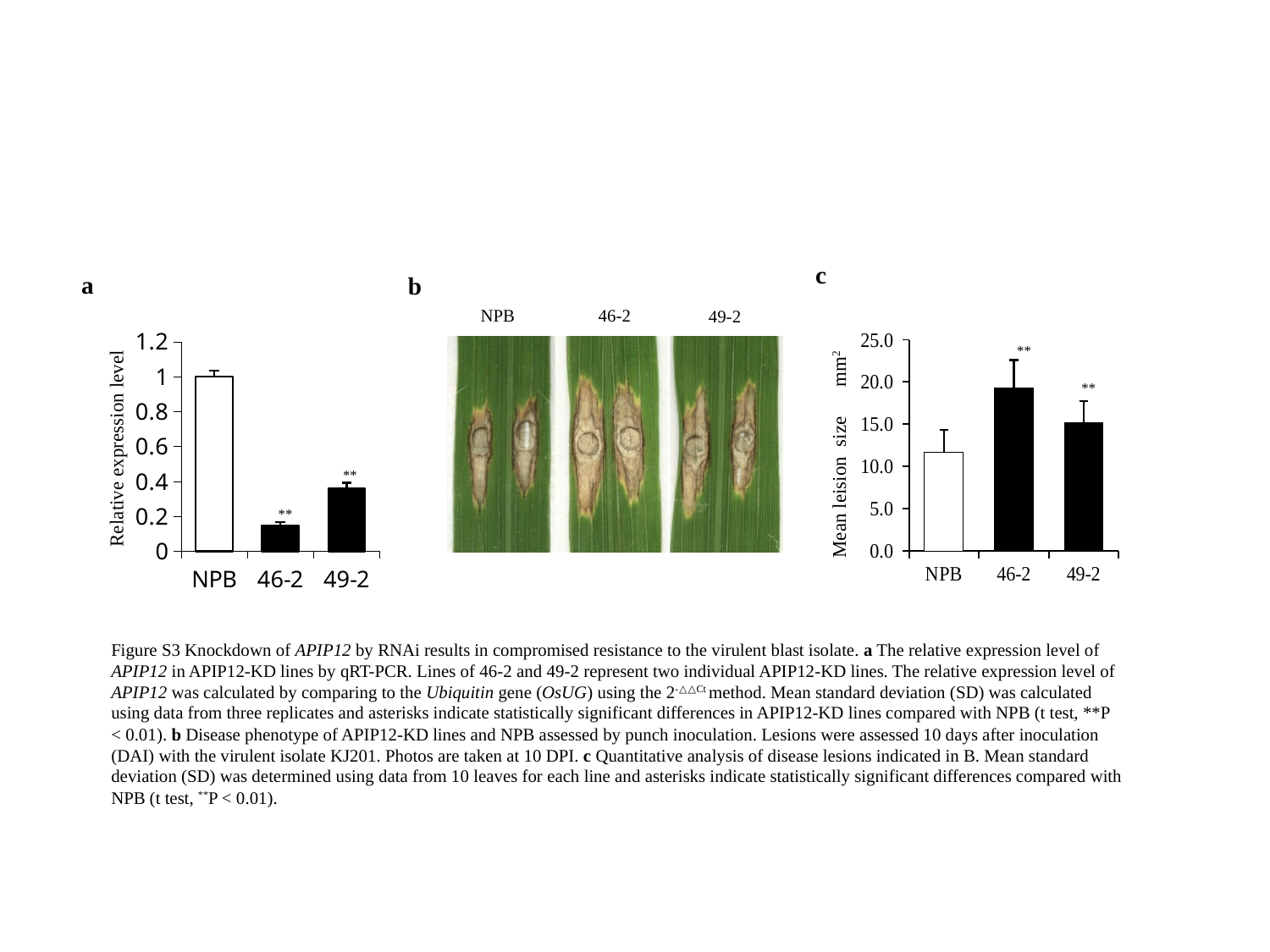

c
**
Mean leision size （mm2）
**
### Chart
| Category | |
|---|---|
| NPB | 11.7075 |
| 46-2 | 19.25066666666667 |
| 49-2 | 15.207272727272722 |a
### Chart
| Category | |
|---|---|
| NPB | 1.0 |
| 46-2 | 0.14933 |
| 49-2 | 0.36092000000000024 |Relative expression level
**
**
b
 NPB
 46-2
 49-2
Figure S3 Knockdown of APIP12 by RNAi results in compromised resistance to the virulent blast isolate. a The relative expression level of APIP12 in APIP12-KD lines by qRT-PCR. Lines of 46-2 and 49-2 represent two individual APIP12-KD lines. The relative expression level of APIP12 was calculated by comparing to the Ubiquitin gene (OsUG) using the 2-△△Ct method. Mean standard deviation (SD) was calculated using data from three replicates and asterisks indicate statistically significant differences in APIP12-KD lines compared with NPB (t test, **P < 0.01). b Disease phenotype of APIP12-KD lines and NPB assessed by punch inoculation. Lesions were assessed 10 days after inoculation (DAI) with the virulent isolate KJ201. Photos are taken at 10 DPI. c Quantitative analysis of disease lesions indicated in B. Mean standard deviation (SD) was determined using data from 10 leaves for each line and asterisks indicate statistically significant differences compared with NPB (t test, **P < 0.01).
