# Supplementary material for: The Nup98 Homolog APIP12 Targeted by the Effector AvrPiz-t is Involved in Rice Basal Resistance Against Magnaporthe oryzae
Source: Rice (N Y). 2017 Feb 15;10:5. doi: 10.1186/s12284-017-0144-7 (PMC5311014; doi:10.1186/s12284-017-0144-7)
Supplement: Additional file 4: Figure S4. — Overexpression of APIP12 does not alter the disease resistance to rice blast. (PPTX 117 kb) [file 12284_2017_144_MOESM4_ESM.pptx]

## Slide 1
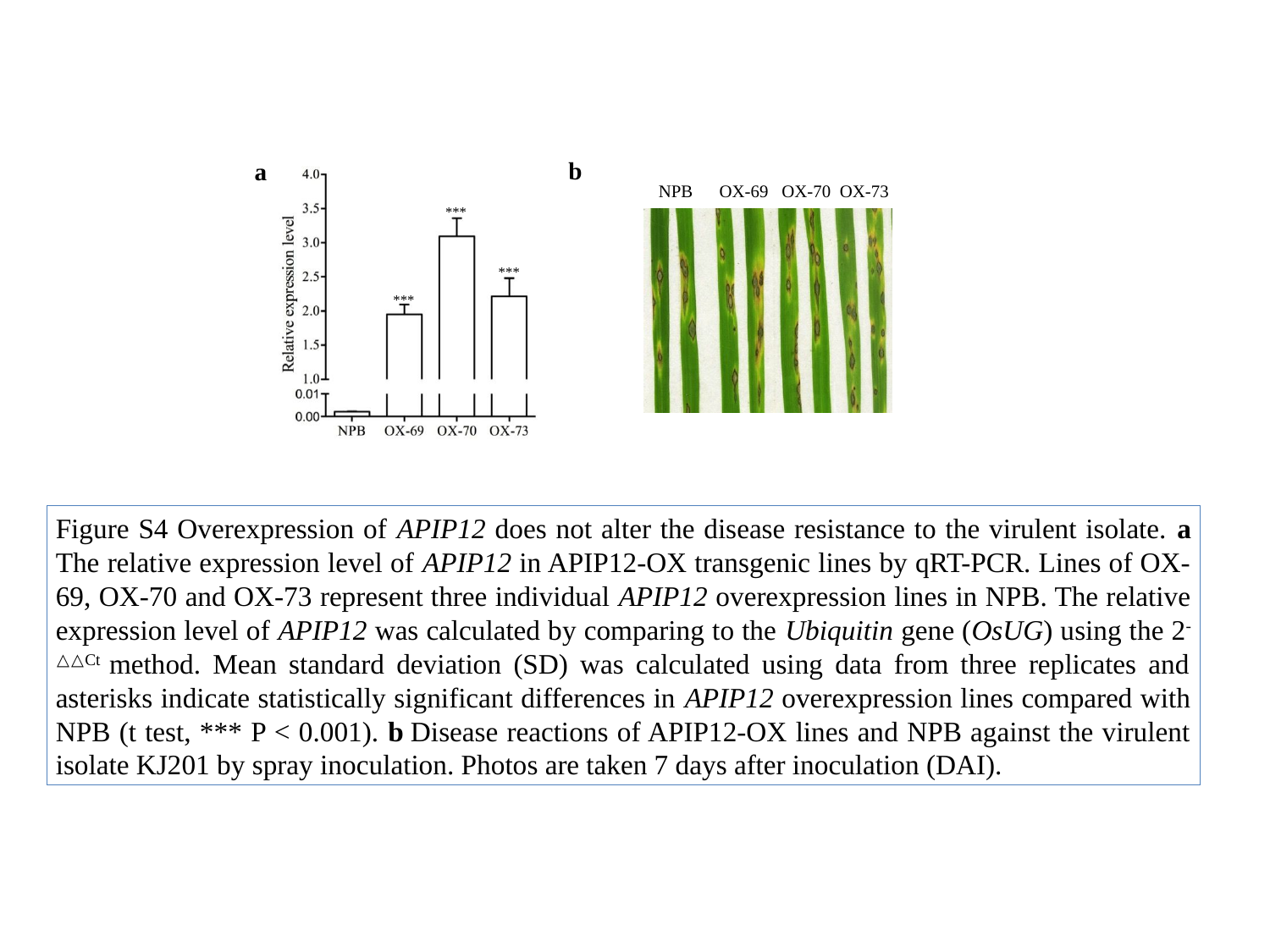

a
***
***
***
b
NPB OX-69 OX-70 OX-73
### Chart
| Category |
|---|Figure S4 Overexpression of APIP12 does not alter the disease resistance to the virulent isolate. a The relative expression level of APIP12 in APIP12-OX transgenic lines by qRT-PCR. Lines of OX-69, OX-70 and OX-73 represent three individual APIP12 overexpression lines in NPB. The relative expression level of APIP12 was calculated by comparing to the Ubiquitin gene (OsUG) using the 2-△△Ct method. Mean standard deviation (SD) was calculated using data from three replicates and asterisks indicate statistically significant differences in APIP12 overexpression lines compared with NPB (t test, *** P < 0.001). b Disease reactions of APIP12-OX lines and NPB against the virulent isolate KJ201 by spray inoculation. Photos are taken 7 days after inoculation (DAI).
