# Supplementary material for: The Nup98 Homolog APIP12 Targeted by the Effector AvrPiz-t is Involved in Rice Basal Resistance Against Magnaporthe oryzae
Source: Rice (N Y). 2017 Feb 15;10:5. doi: 10.1186/s12284-017-0144-7 (PMC5311014; doi:10.1186/s12284-017-0144-7)
Supplement: Additional file 5: Table S1. — Relatively expression level of PR genes in both APIP12-KD (RNAi) and APIP12-KO mutants compared to respective wild type plants at 72 h after infection with the virulent isolate GUY11. (DOC 30 kb) [file 12284_2017_144_MOESM5_ESM.doc]

Table S1. Relatively expression level of *PR* genes in both APIP12-KD (RNAi) and APIP12-KO mutants compared to respective wild type plants at 72 hours after infection with the virulent isolate GUY11. Error bars indicate the SD from three biological replicates (n=3), and P value indicates significant differences between APIP12-knockout mutant and the wild type plant (Student’s T-Test)

| PR gene family | Gene | RNAi/NPB | KO/ZH11 | P value |
| --- | --- | --- | --- | --- |
| PR1 | *Os01g28450* | 0.36±0.015 | 0.45±0.090 | 0.1345 |
| PR2 | *Os01g71340* | 0.65±0.028 | 0.68±0.018 | 0.2580 |
| PR5 | *Os12g43380* | 0.70±0.343 | 0.65±0.150 | 0.8471 |
| PR5 | *Os12g43440* | 0.81±0.029 | 0.59±0.189 | 0.1194 |
| PR10 | *Os12g36880* | 0.38±0.017 | 0.37±0.051 | 0.7082 |
