# Supplementary material for: The Nup98 Homolog APIP12 Targeted by the Effector AvrPiz-t is Involved in Rice Basal Resistance Against Magnaporthe oryzae
Source: Rice (N Y). 2017 Feb 15;10:5. doi: 10.1186/s12284-017-0144-7 (PMC5311014; doi:10.1186/s12284-017-0144-7)
Supplement: Additional file 6: Figure S5. — APIP12 interacts with Nup96 (LOC_Os03g07580) in Y2H assays. (PPTX 69 kb) [file 12284_2017_144_MOESM6_ESM.pptx]

## Slide 1
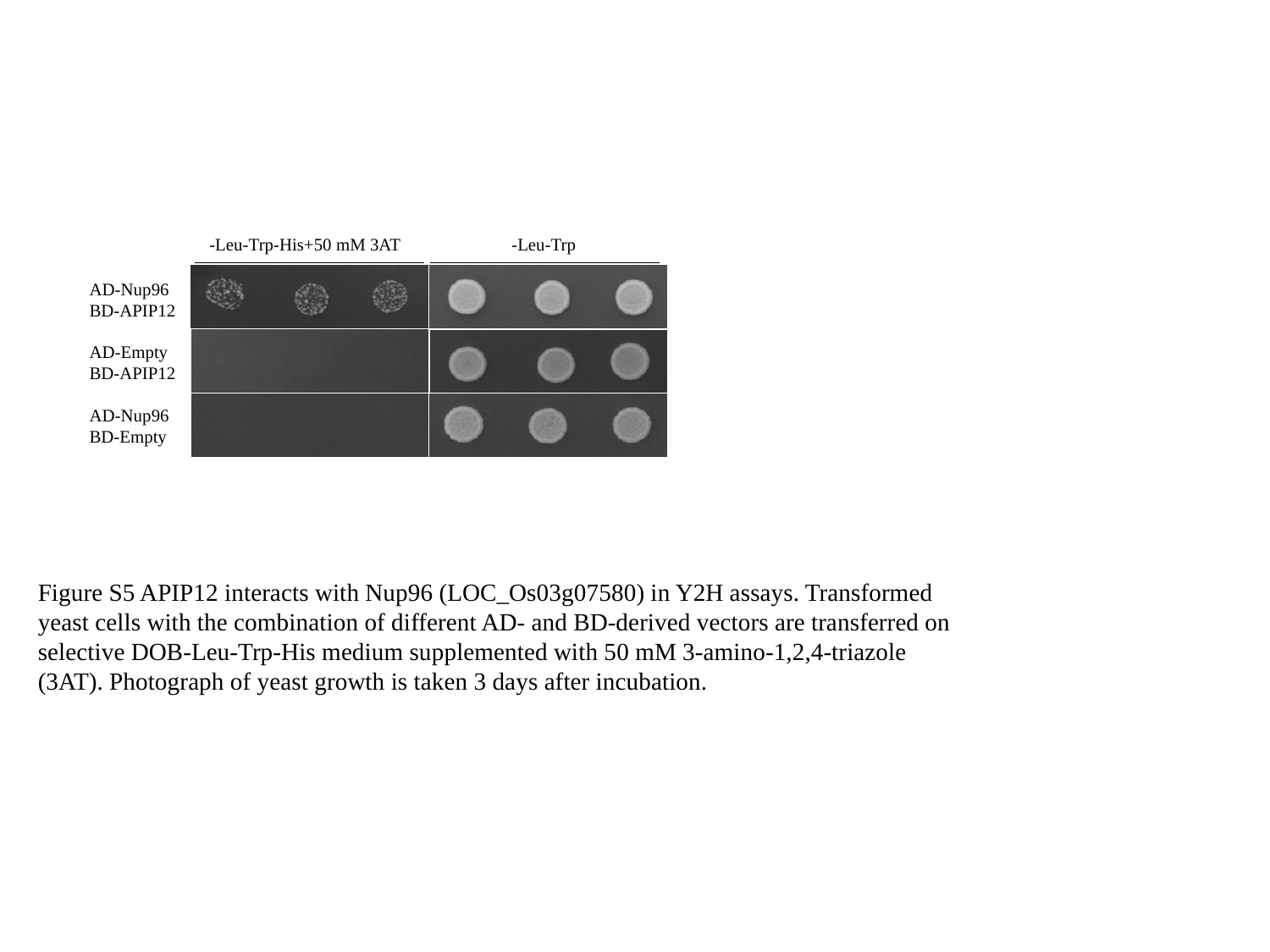

-Leu-Trp-His+50 mM 3AT
-Leu-Trp
AD-Nup96
BD-APIP12
AD-Empty
BD-APIP12
AD-Nup96
BD-Empty
Figure S5 APIP12 interacts with Nup96 (LOC_Os03g07580) in Y2H assays. Transformed yeast cells with the combination of different AD- and BD-derived vectors are transferred on selective DOB-Leu-Trp-His medium supplemented with 50 mM 3-amino-1,2,4-triazole (3AT). Photograph of yeast growth is taken 3 days after incubation.
