# Supplementary material for: The Nup98 Homolog APIP12 Targeted by the Effector AvrPiz-t is Involved in Rice Basal Resistance Against Magnaporthe oryzae
Source: Rice (N Y). 2017 Feb 15;10:5. doi: 10.1186/s12284-017-0144-7 (PMC5311014; doi:10.1186/s12284-017-0144-7)
Supplement: Additional file 7: Table S2. — Primers used in this study. (DOC 69 kb) [file 12284_2017_144_MOESM7_ESM.doc]

| Table S2. Primers used in this study. | | |
| --- | --- | --- |
| Primers | Sequence (5’-3’) | Purpose |
| RNAi F | CGCAGCAAAGTATGCAACAG | Construct RNAi vector of APIP12 |
| RNAi R | CATCGTGGAGTTCAATGAGC |
| APIP12 AscI F | GGCGCGCCATGAGCTCTTCTTCGTCCTG | Construct overexpression vector of APIP12 |
| APIP12 PacI R | TTAATTAACTACATATTGAAATTCTTAACCCTG |
| APIP12 SalI F | GTCGACATGATGAGCTCTTCTTCGTCCTG | Cloning of APIP12 F in pPC86 and pDBLeu |
| APIP12 SpeI R | ACTAGTCTACATATTGAAATTCTTAACCCTG |
| APIP12 314 SpeI R | ACTAGTTTACTGACATGCAAATCCAGTAGAAGG | Cloning of APIP12 N in pPC86 and pDBLeu with APIP12 SalI F |
| APIP12 314 SalI F | GTCGACATGGAAAATGTATTCAGCAACTCAGCGG | Cloning of APIP12 M in pPC86 and pDBLeu |
| APIP12 474 SpeI R | ACTAGTTTAATCGGCGCTGTACAGGCGAGGCAG |
| APIP12 474 SalI F | GTCGACATGTACTACACCGTGCCATCTATTGTG | Cloning of APIP12 C in pPC86 and pDBLeu with APIP12 SpeI R |
| APIP12 EcoRI F | GAATTCATGATGAGCTCTTCTTCGTCCTG | Cloning of GST:APIP12 in pGEX-6P-1 |
| APIP12 SalI R | GTCGACCTACATATTGAAATTCTTAACCCTG |
| APIP12 314 SalI R | GTCGACTTACTGACATGCAAATCCAGTAGAAGG | Cloning of GST:APIP12 N in pGEX-6P-1 with APIP12 EcoRI F |
| APIP12 314 EcoRI F | GAATTCATGGAAAATGTATTCAGCAACTCAG | Cloning of GST:APIP12 M in pGEX-6P-1 |
| APIP12 474 SalI R | GTCGACTTAATCGGCGCTGTACAGGCGAGGCAG |
| Nup96 SalI F | GTCGACATGTCTTCCGACCCGGTGTTCC | Cloning of AD:Nup96 |
| Nup96 SpeI R | ACTAGTTCAGTCCCTGCAAAGTATGTCGGT |
| APIP6-pro F | GCCCGAATTCATGGGTGCGAGGGAGG | Cloning of APIP6 in pMAL-c2x |
| APIP6-pro R | CCCGTCGACCTACATCCTTGGGGTGTGC |
| APIP6-SalI F | GGTCGACCATGGGTGCGAGGGAGGAGGTGAG | Cloning of APIP6 in pPC86 |
| APIP6-SpeI R | ACTAGTCTACATCCTTGGGGTGTGCATTTG |
| Pro F | CGTACGGGCGCTCGATCTGCTTC | Validation of Tos17 insertion |
| Pro R | CTGAAGTTACCACGTTGCTGAAATG |
| TOS-F | ATTGTTAGGTTGCAAGTTAGTTAAG |
| 314 F | CTGACATGCAAATCCAGTAGAAGG | RT-PCR of *APIP12* |
| 414 R | ATGACCACCGGTTGGACTAGATTG |
| *Os*Actin-F | CCTGCTATGTACGTCGCCATC | RT-PCR of rice *Actin* |
| *Os*Actin-R | CCGCAGCTTCCATTCCTATGA |
| 334 F | AATACCATAGATTTGCTACTTCC/ | qRT-PCR of *APIP12* |
| 394 R | TTTGATAATCAGCTCTTGGTTCTC |
| OsUG-F | TTCTGGTCCTTCCACTTTCAG/ | qRT-PCR of rice *Ubiquitin* |
| OsUG-R | ACGATTGATTTAACCAGTCCATGA |
| ZDD154-F | GCTCAAAACTCCCCGCAG/ | qRT-PCR of *Os01g28450* (*PR1* family) |
| ZDD154-R | TTCTCGCCAAGGTTGTTCC |
| ZDD155-F | TGGGTGGACGAGAAGAAGTA | qRT-PCR of *Os01g28500* (*PR1* family) |
| ZDD155-R | AACGATGTTGCCACGGGG |
| ZDD158-F | CGTGGGTGTCGGAGAAGC | qRT-PCR of *Os07g03710* (*PR1* family) |
| ZDD158-R | GCAGGTGATGAAGACGCC |
| ZDD159-F | TTGCGGCCATTCCTACAGT | qRT-PCR of *Os01g71340* (*PR2* family) |
| ZDD159-R | TGGTGAGGGCGATGCTTG |
| ZDD161-F | GTCGCCTCCATCGTGTCG | qRT-PCR of *Os06g51060* (*PR3* family) |
| ZDD161-R | ACCCGCCCGTGGTCTCGT |
| ZDD163-F | GCGACTTCTCCACCCTAC | qRT-PCR of *Os10g39680* (*PR3* family) |
| ZDD163-R | TGGCACGACGGCTTGTTC |
| ZDD164-F | TCGTGGCGTCAGAAGTATGG | qRT-PCR of *Os11g37950* (*PR4* family) |
| ZDD164-R | ACGGTGTCCCAGTCCAGG |
| ZDD165-F | TGGCACAAGAGGCGTCCAA | qRT-PCR of *Os11g37960* (*PR4* family) |
| ZDD165-R | CCACAGAAGGCGGTCCATC |
| ZDD166-F | AACTGGGACCTGAACAAAGT | qRT-PCR of *Os11g37970* (*PR4* family) |
| ZDD166-R | TCTTCACCTGGATACACTTGC |
| ZDD170-F | CGGCAGCCAGGACTTCTA | qRT-PCR of *Os12g43380* (*PR5* family) |
| ZDD170-R | GCAGAAGACGACTTGGTAG |
| ZDD171-F | GTACAACGTCGCCATGAGC | qRT-PCR of *Os12g43440* (*PR5* family) |
| ZDD171-R | GCAGAAGACGACTCGGTAG |
| ZDD175-F | GTCTGCGCCGGATTCATC | qRT-PCR of *Os12g36850* (*PR10* family) |
| ZDD175-R | AACCTCCAACACCTCAACCT |
| ZDD177-F | AGTCATGTCCTAAAGTCGGA | qRT-PCR of *Os12g36880* (*PR10* family) |
| ZDD177-R | GCCATAGTAGCCATCCACG |
